# Use of AUDIT, and measures of drinking frequency and patterns to detect associations between alcohol and sexual behaviour in male sex workers in Kenya

**DOI:** 10.1186/1471-2458-11-384

**Published:** 2011-05-25

**Authors:** Stanley Luchters, Scott Geibel, Masila Syengo, Daniel Lango, Nzioki King'ola, Marleen Temmerman, Matthew F Chersich

**Affiliations:** 1 International Centre for Reproductive Health (ICRH), Department of Obstetrics and Gynaecology, Ghent University, Belgium; 2 Population Council, Nairobi, Kenya; 3 International Centre for Reproductive Health (ICRH), Mombasa, Kenya; 4 Centre for Health Policy, School of Public Health, Faculty of Health Sciences, The University of Witwatersrand, South Africa

**Keywords:** alcohol, sexual behaviour, Kenya, indicators, men who have sex with men, sex worker

## Abstract

**Background:**

Previous research has linked alcohol use with an increased number of sexual partners, inconsistent condom use and a raised incidence of sexually transmitted infections (STIs). However, alcohol measures have been poorly standardised, with many ill-suited to eliciting, with adequate precision, the relationship between alcohol use and sexual risk behaviour. This study investigates which alcohol indicator - single-item measures of frequency and patterns of drinking ( > = 6 drinks on 1 occasion), or the Alcohol Use Disorders Identification Test (AUDIT) - can detect associations between alcohol use and unsafe sexual behaviour among male sex workers.

**Methods:**

A cross-sectional survey in 2008 recruited male sex workers who sell sex to men from 65 venues in Mombasa district, Kenya, similar to a 2006 survey. Information was collected on socio-demographics, substance use, sexual behaviour, violence and STI symptoms. Multivariate models examined associations between the three measures of alcohol use and condom use, sexual violence, and penile or anal discharge.

**Results:**

The 442 participants reported a median 2 clients/week (IQR = 1-3), with half using condoms consistently in the last 30 days. Of the approximately 70% of men who drink alcohol, half (50.5%) drink two or more times a week. Binge drinking was common (38.9%). As defined by AUDIT, 35% of participants who drink had hazardous drinking, 15% harmful drinking and 21% alcohol dependence. Compared with abstinence, alcohol dependence was associated with inconsistent condom use (AOR = 2.5, 95%CI = 1.3-4.6), penile or anal discharge (AOR = 1.9, 95%CI = 1.0-3.8), and two-fold higher odds of sexual violence (AOR = 2.0, 95%CI = 0.9-4.9). Frequent drinking was associated with inconsistent condom use (AOR = 1.8, 95%CI = 1.1-3.0) and partner number, while binge drinking was only linked with inconsistent condom use (AOR = 1.6, 95%CI = 1.0-2.5).

**Conclusions:**

Male sex workers have high levels of hazardous and harmful drinking, and require alcohol-reduction interventions. Compared with indicators of drinking frequency or pattern, the AUDIT measure has stronger associations with inconsistent condom use, STI symptoms and sexual violence. Increased use of the AUDIT tool in future studies may assist in delineating with greater precision the explanatory mechanisms which link alcohol use, drinking contexts, sexual behaviours and HIV transmission.

## Background

Globally, researchers and policy makers are increasingly giving attention to the effects of alcohol use on sexual behaviour; with many making a strong case that heavy alcohol use is an important cause of unsafe sexual behaviour and consequent HIV transmission in sub-Saharan Africa [1-3]. Most, but not all studies [4-6], have linked alcohol use with an increased number of sexual partners, regretted sexual relations, condom accidents and an increased incidence of sexually transmitted infections (STI) [7-9]. Studies in sub-Saharan Africa, in particular, have found strong associations between alcohol consumption and unprotected sex, early sexual debut, concurrent partners and having HIV [10-13]. A few studies in this setting have also identified strong linkages between unsafe sex and the use of alcohol in combination with other substances [14-16].

Measuring the consequences of alcohol use is complex as the harms of alcohol depend on the total volume consumed, the way it is drunk (drinking patterns) and drinking contexts [17,18]. A large number of indicators have been devised to measure this complexity - the WHO Global Information System on Alcohol and Health gathers information on more than 200 alcohol indicators [18]. Many of these indicators are self-reported and these substantially underestimate the volume of alcohol consumed [19]. Early studies on alcohol and sexual behaviour in Africa used a wide range of alcohol measures and were mostly secondary reports of studies designed for other purposes [11]. Poor standardisation of indicators, with a wide range or undefined length of the recall period, has hampered investigation of the effects of drinking behaviours on sexual behaviour. For example, a meta-analysis of associations between alcohol and HIV resorted to using a binary exposure variable (alcohol use: yes or no), irrespective of recall reference period or whether the indicator measured alcohol volume or pattern, for example [11]. More nuanced differentiation of alcohol usage and its consequences will facilitate a more definitive assessment of the causal pathways between alcohol and sexual behaviour, and potentially guide formulation of interventions to mitigate these effects.

In sub-Saharan Africa, a few studies have documented associations between alcohol and unsafe sex in men who have sex with men [20-22] and in male sex workers [23]. A study in South Africa found that three quarters of men who have sex with men had problem drinking and more than half had 10 or more drinks on a typical drinking day [20]. A 2006 survey of male sex workers who sell sex to men in Mombasa Kenya found that frequent alcohol use (drinking more than two days a week) was associated with unprotected anal sex with male clients [23], a well recognised risk factor for HIV transmission [23,24]. Other dimensions of alcohol use, however, were not measured. Here, in a follow-up to the 2006 Mombasa survey, we investigate which indicator of alcohol use - single-item commonly used measures of frequency and patterns of drinking, or the WHO Alcohol Use Disorders Identification Test (AUDIT) [25] - is associated with unsafe sexual behaviour. In addition to comparing the validity of the three psychometric instruments, the study also documents the burden of harmful alcohol use in this population and its association with sexual behaviours.

## Methods

This cross-sectional survey was conducted in 2008, using the same methods as a study in 2006, which was reported previously [23]. In brief, study participants were recruited from 65 venues in the Mombasa district including nightclubs, beach areas or bars, streets, parks, and private brothels or businesses. Sampling was done with a probability proportional to the population size at each venue. This was based on a sampling frame derived from a mapping of venues and a previous enumeration of the study population [26]. Trained peer workers identified and recruited consecutive men until reaching the pre-specified quota for that venue. Eligible respondents were aged 16 years or older, and men who currently sell sex to men in exchange for money or goods. Men consenting to participate were accompanied to a central location for a structured interview, done by trained interviewers using handheld computers (Dell Axim ×3 handheld computers with Perseus Mobile Survey 7 software, Perseus Development Corporation, MA, USA). In the period between the two surveys, peer educators were trained to provide on counselling interventions on alcohol and drug use, and sexual risk behaviour (five-days training and a follow-up workshop). They delivered these interventions as part of a broader HIV prevention package.

Respondents were given free condoms, water-based lubrication and 300 Kenya Shillings (about US $4.50) for their time and return transport. Information was provided about local clinics, including a research centre which provides specialised services for male sex workers, including treatment for anal STIs. Participants with alcohol dependence were referred to a local alcohol counselling facility and for treatment at a hospital-based clinic. The Kenyatta National Hospital Ethics and Research Committee approved the study activities, as did the Population Council's Institutional Review Board. The Coast Provincial Medical Office authorized the study, and gave a letter of explanation and justification for the survey activity. All participants provided informed consent and no personal identifiers were recorded on study documents.

### Study variables and data management

Study variables were grouped into the following categories: socio-demographic characteristics, use of alcohol and other substances, sexual behaviours, violence and STI symptoms. Respondents were asked whether they lived alone, with a man or a woman. They provided information on their duration of sex work and whether this constituted part or full-time employment. For the purposes of assessing whether age and duration of sex work were potential confounding variables, these variables were dichotomized above, or at and below their median values.

Alcohol behaviours were assessed using AUDIT, which was previously validated in Kenya [27]. This tool contains 10 items each scored 0-4. Current drinkers were defined as men who reported drinking weekly, or less than or equal to once per month. Total scores were categorised as alcohol abstainers (total score 0), low-risk drinking (total score 1-7), hazardous drinking (total score 8-15), harmful drinking (total score 16-19) and alcohol dependence (total score 20-40). The recall reference period varies considerably between the 10 AUDIT items, from weekly, monthly, and even yearly for items such as injury of self or others in past year. As the categories harmful drinking and alcohol dependence are often combined in the AUDIT tool to form a single category called harmful drinking, we analysed this variable with these categories separated and then combined [25]. Two single-item measures of alcohol use were also collected: frequency of use (abstinence; drinking four or less times a month; and two or more times per week) and pattern of use (binge drinking of six or more drinks on one occasion in the past month or less frequently). The comparator group for each of the three alcohol measures was alcohol abstainers. This consisted of men who responded never when asked "How often do you have a drink containing alcohol?". Ever use of Khat (*Catha edulis *, an amphetamine-like stimulant containing an alkaloid called cathinone); rohypnol (flunitrazepam, a short-intermediate acting benzodiazepine); and heroin or cocaine were also assessed.

To assess associations between the three measures of alcohol use and sexual behaviour, three dependant variables were selected *a priori *, namely: inconsistent condom use in anal sex with male clients in the past 30 days; experience of sexual assault or rape in the past 12 months; and penile or anal discharge in the past 12 months. Symptoms of anal or penile discharge were considered indicators of having an STI, most likely *Neisseria gonorrhoeae *or *Chlamydia *[28].

### Data analysis

Data were analyzed using Intercooled Stata version 8 (StataCorp, College Station, TX, USA). Multivariable models were constructed for each of the three dependant variables. Firstly, chi-square tests were used to detect differences between potential confounders and the alcohol measures (AUDIT groups, frequency and pattern). For each of the potential confounding factors (variables shown in Table 1), there was substantive biological plausibility or previous evidence showing an association between the variable and both alcohol exposure and the three outcomes assessed. Potential confounding factors associated with the outcome and exposure (*P *<0.10) were included in multivariate logistic regression models assessing the relation between the three alcohol behaviour indicators and the three dependent variables. Using stepwise forward-fitting logistic regression, potential confounders were included in the model beginning with the covariate with the lowest *P *value [29]. Variables that did not markedly alter the model fit were removed from the model. Where interaction was considered biologically plausible, variables were evaluated for effect modification using a likelihood ratio test. Adjusted odds ratios were presented based on the final multivariable models.

**Table 1:** Characteristics of male sex workers in Mombasa Kenya and associations between characteristics and AUDIT scores, alcohol frequency and drinking patterns

Variable	Alcohol abstainers n (%)	Audit scores n (%)					Frequency of alcohol use n (%)			Pattern of alcohol use n (%)		
		
		Audit score 1-7	Audit score 8-15	Audit score 16-19	Audit score 20-40	*P *	4 or less times per month	2 or more times per week	*P *	Non binge drinker	Binge (≥6 alcohol units)	*P *
Age ≤ 23 years	69 (51.1)	49 (55.1)	55 (51.9)	18 (38.3)	31 (47.7)	0.43	71 (46.7)	82 (52.9)	0.54	76 (56.3)	77 (44.8)	0.13

Education level None or incomplete primary Primary or incomplete secondary Secondary or higher	45 (33.3) 66 (48.9) 24 (17.8)	29 (32.6) 40 (44.9) 20 (22.5)	30 (28.3) 53 (50.0) 23 (21.7)	13 (27.7) 28 (59.6) 6 (12.8)	17 (26.2) 29 (44.6) 19 (29.2)	0.51	41 (27.0) 81 (53.3) 30 (19.7)	48 (31.0) 69 (44.5) 38 (24.5)	0.42	44 (32.6) 63 (46.7) 28 (20.7)	45 (26.2) 87 (50.6) 40 (23.3)	0.57

Religion Catholic Protestant Muslim Animist or none	34 (25.2) 21 (15.6) 76 (56.3) 4 (3.0)	17 (19.1) 17 (19.1) 49 (55.1) 6 (6.7)	28 (26.4) 12 (11.3) 56 (52.8) 10 (9.4)	9 (19.2) 8 (17.0) 26 (55.3) 4 (8.5)	25 (39.1) 12 (18.8) 25 (39.1) 2 (3.1)	0.13	34 (22.4) 25 (15.6) 87 (57.2) 6 (4.0)	45 (29.2) 24 (16.5) 69 (44.8) 16 (10.4)	0.053	28 (20.7) 22 (16.3) 76 (56.3) 9 (6.7)	51 (29.8) 27 (15.8) 80 (46.8) 13 (7.6)	0.30

Lives with a male or female partner	40 (29.6)	27 (30.3)	28 (26.4)	14 (29.8)	22 (33.9)	0.89	46 (30.3)	45 (29.0)	0.97	36 (26.7)	55 (32.0)	0.60

Part-time sex work	76 (56.3)	49 (55.1)	59 (55.7)	22 (46.8)	30 (46.2)	0.56	89 (58.6)	71 (45.8)	0.059	68 (50.4)	92 (53.5)	0.62

Years of sex work 6 or more years	50 (38.5)	45 (51.1)	52 (49.5)	32 (68.1)	33 (51.6)	**0.011 **	76 (50.7)	86 (55.8)	**0.012 **	63 (47.0)	99 (58.2)	**0.003 **

Number of partners (past week^¥ ^) 3 or more clients 1 or more non-paying partners	43 (31.9) 45 (33.6)	25 (28.1) 27 (30.3)	38 (36.2) 32 (30.2)	15 (31.9) 13 (27.7)	23 (35.9) 27 (41.5)	0.77 0.49	36 (23.7) 45 (29.6)	65 (42.5) 54 (34.8)	**0.002 ** **0.60 **	42 (31.1) 35 (25.9)	59 (34.7) 64 (37.2)	0.78 0.11

Role in anal sex with last male client Insertive only Receptive only Both insertive and receptive	72 (53.7) 55 (41.0) 7 (5.2)	38 (44.2) 35 (40.7) 13 (15.1)	60 (56.6) 31 (29.3) 15 (14.2)	24 (51.1) 20 (42.6) 3 (6.4)	29 (44.6) 28 (43.1) 8 (12.3)	0.11	75 (50.0) 57 (38.0) 18 (12.0)	76 (49.4) 57 (37.0) 21 (13.6)	0.20	62 (47.0) 48 (36.4) 22 (16.7)	89 (51.7) 66 (38.4) 17 (9.9)	0.052

Prevention knowledge: aware that^¥ ^ HIV can be transmitted via anal sex Consistent condoms prevent HIV	98 (72.6) 121 (89.6)	68 (76.4) 83 (93.3)	80 (75.5) 97 (91.5)	32 (68.1) 38 (80.9)	46 (70.8) 56 (86.2)	0.85 0.26	116 (76.3) 138 (90.8)	110 (71.0) 136 (87.7)	0.73 0.84	101 (74.8) 128 (94.8)	125 (72.7) 146 (84.9)	0.86 **0.026 **

Ever tested for HIV	83 (61.9)	64 (71.9)	66 (62.3)	30 (63.8)	40 (61.5)	0.56	97 (63.8)	103 (66.5)	0.72	88 (65.2)	112 (65.1)	0.81

Substance use^¥ ^ Khat Cocaine or heroin Rohypnol	91 (67.4) 4 (3.0) 10 (7.4)	64 (71.9) 7 (7.9) 7 (7.9)	87 (82.1) 11 (10.4) 19 (17.9)	42 (89.4) 5 (10.6) 11 (23.4)	50 (76.9) 7 (10.8) 19 (29.2)	**0.012 ** 0.15 **< 0.001 **	112 (73.7) 11 (7.2) 22 (14.5)	131 (84.5) 19 (12.3) 34 (21.9)	**0.003 ** **0.012 ** **0.002 **	98 (72.6) 12 (8.9) 18 (13.3)	145 (84.3) 18 (10.5) 38 (22.1)	**0.002 ** **0.041 ** **0.001 **

Total cases (n)	135	89	106	47	65		152	155		135	172	

¥ Multiple-response question; men who abstain from alcohol (column 2) were included in each comparison

## Results

### Population description and sexual behaviour

A total of 516 eligible men were identified, of whom 442 enrolled in the study. Participants were a mean 24.6 years (standard deviation [sd] = 5.2 years) and nearly all (98.2%; data not in table) said they were from Kenya. The mean age at which the respondents began sex work was 18.3 years (sd = 4.0 years). Having been married to a woman was not uncommon, reported by nearly a quarter of men (22.6%), while 14.0% presently live with a female sex partner (data not in table). Over half were Muslim (52.6%), followed by Catholics (25.6%) and Protestants (15.9%). The population was about equally divided between part-and full-time sex workers. Table 1 presents descriptions of the population and their sexual behaviour.

In the past year, a substantial number reported having been physically assaulted because someone believed they had sex with men (13.9%; data not in table), while 11.8% had experienced sexual violence (sexual assault or rape) in the same period. About a quarter of men were not aware that HIV can be transmitted through anal sex.

In the past week participants reported a median 2 male clients (IQR: 1-3), while 22.7% had one non-paying partner and 10.0% had two or more (data not in table). Only half had used condoms with each sex act in the last 30 days with paying clients, and fewer with non-paying partners (39.5%; data not in table). With the last male client, most men had insertive anal sex only (50.9%), while 10.5% reported both insertive and receptive anal intercourse.

Nearly a quarter of men reported having penile or anal discharge in the past year, with no difference detected between men who abstained from alcohol (21.5%) and those who drank (22.5%). Over the same recall period, a similar proportion had a penile or anal ulcer (22.6%) while 31.4% reported burning on urination (data not in table).

### Substance use

Nearly 70% of men reported currently drinking alcohol and they had a longer duration of sex work than alcohol abstainers (median 6 years, IQR = 3-9 versus 5 years, IQR = 3-7 years; *P *= 0.03; data not in table). Based on the AUDIT measure, 29.0% of the men who drink had low-risk alcohol use, 34.5% hazardous drinking, 15.3% harmful drinking, and 21.2% had alcohol dependence (characteristics of alcohol use are presented in Table 2). Half of non-abstainers reported drinking two or more times per week. Binge drinking was common (38.9%), and occurred frequently, with 37.2% of these men reporting two or more binge drinking episodes per week.

**Table 2:** AUDIT scores, frequency of alcohol use and drinking patterns among 442 male sex workers in Mombasa, Kenya

Measures of alcohol use	n (%)
**AUDIT scores **	
Score 0 (alcohol abstinence) Score 1-7 (low-risk drinkers) Score 8-15 (hazardous drinkers) Score 16-19 (harmful drinkers) Score 20-40 (alcohol dependence)	135 (30.5) 89 (20.1) 106 (24.0) 47 (10.6) 65 (14.7)

**Frequency of alcohol use **	
Alcohol abstinence 4 or less times per month 2 or more times per week	135 (30.5 152 (49.5) 155 (50.5)

**Pattern of alcohol use **^	
Alcohol abstinence Non-binge drinking Monthly binge or less Binge 2 to 4 times a month Binge more than 2 times a week	135 (30.5) 135 (30.5) 57 (12.9) 51 (11.5) 64 (14.5)

^ Binge drinking defined as 6 or more drinks on 1 occasion

Lifetime use of other substances was more common among men who drink than those who abstain: drinkers were 1.8 times more likely than abstainers to use khat; (95%CI OR = 1.2-2.9; *P *= 0.008), 3.5 fold more likely to use cocaine or heroin (95%CI OR = 1.2-10.4; *P *= 0.01); and 2.8 times more likely to use rohypnol (95%CI OR = 1.4-5.7; *P *= 0.003; these OR not in table). In multivariate models that contained alcohol indicators and rohypnol use (Table 3), rohypnol was independently associated with inconsistent condom use and sexual violence. In the multivariate models that included AUDIT and rohypnol, inconsistent condom use was 1.7 times more likely in those using rohypnol (95%CI AOR = 1.0-3.1; *P *= 0.049) and sexual violence 2.4 fold more likely (95%CI AOR = 1.1-4.8; *P *= 0.017; these AOR not in table). No interaction was detected between alcohol use and use of khat, cocaine or heroin, or rohypnol in any of the models.

**Table 3:** Multivariate analysis of associations between alcohol measures and condom use, sexual violence and sexually-transmitted infections in male sex workers

Variable	Risk factor	n (%)	Crude odds ratio (95% CI)	*P *	Adjusted odds ratio (95% CI)	*P *
**Inconsistent condom use with clients**	Alcohol abstainers (comparison group)*	58 (43.0)	1.0		1.0	
						
	**Audit assessment**					
	Audit score 1-7 (low-risk drinkers)	45 (50.6)	1.36 (0.79-2.33)		1.33 (0.77-2.28)	
	Audit score 8-15 (hazardous drinkers)	48 (45.3)	1.10 (0.66-1.84)		1.06 (0.63-1.78)	
	Audit score 16-19 (harmful drinkers)	25 (53.2)	1.50 (0.77-2.95)		1.37 (0.70-2.70)	
	Audit score 20-40 (alcohol dependence)	44 (67.7)	2.78 (1.47-5.28)	**0.004**	2.46 (1.31-4.64)	**0.017^**
	
	**Frequency of alcohol use**					
	4 or less times per month	67 (44.1)	1.05 (0.66-1.67)		1.06 (0.65-1.72)	
	2 or more times per week	95 (61.3)	2.10 (1.30-3.39)	**0.002**	1.83 (1.12-2.98)	**0.013^%^**
	
	**Pattern of alcohol use**					
	Non-binge drinkers	65 (48.2)	1.23 (0.76-1.99)		1.19 (0.73-1.93)	
	Binge drinkers	97 (56.4)	1.72 (1.08-2.72)	**0.018**	1.58 (0.99-2.51)	0.051^

**Sexual violence** **(rape or sexual assault)**	Alcohol abstainers (comparison group)*	13 (9.6)	1.0		1.0	
						
	**Audit assessment**					
	Audit score 1-7 (low-risk drinkers)	7 (7.9)	0.80 (0.31-2.10)		0.74 (0.28-1.96)	
	Audit score 8-15 (hazardous drinkers)	11 (10.5)	1.10 (0.47-2.57)		0.73 (0.30-1.79)	
	Audit score 16-19 (harmful drinkers)	6 (12.8)	1.37 (0.49-3.86)		0.98 (0.33-2.85)	0.13^# ^
	Audit score 20-40 (alcohol dependence)	15 (23.1)	2.82 (1.23-6.44)	**0.010**	2.04 (0.86-4.87)	
	
	**Frequency of alcohol use**					
	4 or less times per month	18 (11.9)	1.27 (0.60-2.71)		1.02 (0.47-2.23)	
	2 or more times per week	21 (13.6)	1.47 (0.70-3.07)	0.30	0.99 (0.46-2.15)	0.98^# ^
	
	**Pattern of alcohol use**					
	Non-binge drinkers	13 (9.6)	1.00 (0.44-2.25)		0.85 (0.37-1.93)	
	Binge drinkers	26 (15.2)	1.68 (0.83-3.43)	0.12	1.14 (0.54-2.41)	0.67^#^

**Self-reported STI ** **(anal or penile discharge)**	Alcohol abstainers (comparison group)*	29 (21.5)	1.0		1.0	
						
	**Audit assessment**					
	Audit score 1-7 (low-risk drinkers)	16 (18.0)	0.80 (0.41-1.58)		0.74 (0.37-1.48)	
	Audit score 8-15 (hazardous drinkers)	20 (18.9)	0.85 (0.45-1.61)		0.90 (0.48-1.72)	
	Audit score 16-19 (harmful drinkers)	15 (31.9)	1.71 (0.81-3.61)		1.67 (0.80-3.52)	**0.019 **|
	Audit score 20-40 (alcohol dependence)	23 (35.4)	2.00 (1.03-3.88)	**0.018**	1.94 (1.01-3.75)	
	
	**Frequency of alcohol use**					
	4 or less times per month	40 (26.3)	1.31 (0.75-2.26)		1.26 (0.72-2.18)	
	2 or more times per week	34 (21.9)	1.03 (0.59-1.80)	0.96	1.05 (0.60-1.84)	0.90|
	
	**Pattern of alcohol use**					
	Non-binge drinkers	29 (21.5)	1.00 (0.56-1.79)		0.98 (0.55-1.77)	
	Binge drinkers	45 (26.2)	1.30 (0.76-2.21)	0.32	1.29 (0.75-2.20)	0.33|

*Alcohol abstainers are the baseline group for all models. ^ adjusted for age & rohypnol use;% adjusted for age, no. of clients & rohypnol use; # adjusted for age, duration of sex work & rohypnol use; | adjusted for age. Age was included as a continuous variable in multivariable analysis

### Association between measures of alcohol use and sexual risk behaviours

Table 3 shows findings of multivariate analysis of associations between alcohol use and condom use, violence and STIs. With the AUDIT measure, men with low-risk, hazardous or harmful alcohol use did not have an increased odds of reporting risk behaviour compared to alcohol abstainers. Male sex workers with alcohol dependence, however, were more likely than abstainers to have inconsistent condom use (AOR = 2.5, 95%CI = 1.3-4.6); anal or penile discharge (AOR = 1.9, 95%CI = 1.0-3.8); and a two-fold higher odds of having experienced sexual violence (though not significant). Men in this group were also more likely to report burning on urination in the past 12 months (OR = 2.5, 95% CI = 1.3-4.6; *P = *0.004, data not in table). Analysis of the same outcomes, but combining the harmful and dependent categories of AUDIT showed similar findings to analysis of dependent drinking alone.

Frequency of alcohol use was also associated with inconsistent condom use (AOR compared to abstainers = 1.8, 95%CI = 1.1-3.0), but not with sexual violence or STI symptoms. Men who drink two or more times a week were 2.4 fold as likely to have 3 or more clients a week than less frequent drinkers (95%CI OR = 1.4-3.9; *P *< 0.001). For the binge drinking indicator, an association was only detected between this variable and inconsistent condom use (AOR = 1.6, 95%CI = 0.99-2.5).

## Discussion

Compared with indicators of drinking frequency or pattern, the AUDIT measure performed better at identifying male sex workers with inconsistent condom use, STI symptoms or sexual violence history. The odds of each study outcome increased markedly in the nearly one in seven men classified as alcohol dependant with the AUDIT tool. A further 10% had harmful drinking, which was similarly associated with the study outcomes. High levels of binge drinking in this study concur with drinking patterns in sub-Saharan Africa which are characterised by drinking to intoxication in public spaces, heavy drinking on weekends and drinking outside of mealtimes [30-33]. Rohypnol use, reported by about 15% of men, was associated with unsafe sex. This warrants examination in future studies, which must carefully select indicators able to further explore this association.

The findings of this study could inform selection and standardization of alcohol indicators in future studies examining the putative causal pathways between alcohol and sexual behaviour. Use of AUDIT may offer greater precision in delineating the complex associations between alcohol use and sexual risk behaviour. Finding that AUDIT had a higher construct validity than conventional measures is, however, not unexpected. Reliability and by extension predictability of psychometric tests is generally correlated with the number of items in a scale. Similarly, the response format of AUDIT questions (categories from 0-4) is likely to have higher discriminant validity than force-choice binary variables such as binge drinking.

Links between alcohol use and selling sex likely account for the association between frequency of drinking and number of clients per week: the more time a sex worker spends in drinking venues, the more clients they obtain, and the higher the likelihood of the person being a full-time sex worker. The frequency of drinking was slightly lower in this survey than in 2006 (more than a half drank on two or more days a week in 2006 [23] while only a third reported this in 2008). Based on existing evidence, the extent of these changes is congruent with the anticipated effect size of alcohol counselling interventions provided by a peer worker [34,35]. However, the design of this study (non-experimental pre-and post-intervention surveys) makes it difficult to distinguish between the effects of the intervention and other intercurrent changes which may have occurred in the population over the study period [36].

This study is limited by the absence of event-level measures (measuring drinking around the time of sex or negotiation of paid sex). These measures allow for more direct testing of the hypothesis as to whether sexual risks coincide with drinking [10,37]. A further limitation is the use of alcohol abstainers as the baseline comparator group in this study. Though most studies show that people with high levels of alcohol consumption have increased sexual risk taking compared to those with lower drinking levels,[10] there is no defined lower limit of alcohol consumption which is considered 'safe' for sexual behaviour. Lack of a known lower safe limit necessitates the use of alcohol abstinent men as the baseline comparator group rather than low-risk drinkers. The alternative, use of low-risk drinkers as a baseline group would, however, be a more realistic measure of the alcohol burden which is modifiable, given that low-risk drinking is the major public health goal for most alcohol reduction interventions [38].

## Conclusion

In conclusion, a quarter of male sex workers who sell sex to men had harmful drinking or alcohol dependence, suggesting a large unmet needs for services. There are few alcohol treatment centres in this setting and provision of Brief Interventions, for example, is limited. Male sex workers in this setting and elsewhere in Africa require targeted alcohol harm reduction interventions, integrated within HIV prevention and treatment services [21,39-41]. In future studies, standardisation of alcohol indicators will improve the ability to identify the intervening explanatory mechanisms between alcohol use and HIV infection [10,42]. At a minimum, alcohol measures should distinguish between alcohol use, harm and dependence, all assessed within the same time frame as the sexual behaviour measures.

## Competing interests

The authors declare that they have no competing interests.

## Authors' contributions

SL, SG and MC conceived the study. SG, SL, MS, DL and NK were responsible for overseeing the study procedures and conduct. MT provided overall study oversight. SL developed the first draft of the article, and all co-authors provided input and assisted in finalizing the paper. All authors read and approved the final version.

## Pre-publication history

The pre-publication history for this paper can be accessed here:

http://www.biomedcentral.com/1471-2458/11/384/prepub

## References

[B1] Hendershot CS George WH Alcohol and sexuality research in the AIDS era: trends in publication activity, target populations and research design AIDS Behav 2007 11 2 217 226 10.1007/s10461-006-9130-6 16897352 PMC2746265

[B2] Parry C Rehm J Poznyak V Room R Alcohol and infectious diseases: an overlooked causal linkage? Addiction 2009 104 3 331 332 19207335 10.1111/j.1360-0443.2008.02500.x

[B3] Chersich MF Rees HV Causal links between binge drinking patterns, unsafe sex and HIV in South Africa: its time to intervene Int J STD AIDS 2010 21 1 2 7 10.1258/ijsa.2000.009432 20029060

[B4] Woolf-King SE Maisto SA Alcohol Use and High-Risk Sexual Behavior in Sub-Saharan Africa: A Narrative Review Arch Sex Behav 2009 10.1007/s10508-009-9516-4 19705274

[B5] Morrison DM Gillmore MR Hoppe MJ Gaylord J Leigh BC Rainey D Adolescent drinking and sex: findings from a daily diary study Perspect Sex Reprod Health 2003 35 4 162 168 10.1363/3516203 12941648

[B6] Cooper LM Does Drinking Promote Risky Sexual Behavior? A Complex Answer to a Simple Question Current directions in psychological science 2006 15 1 19 23 10.1111/j.0963-7214.2006.00385.x

[B7] Bryant KJ Expanding research on the role of alcohol consumption and related risks in the prevention and treatment of HIV/AIDS Subst Use Misuse 2006 41 10-12 1465 1507 10.1080/10826080600846250 17002990

[B8] Carlson SR Johnson SC Jacobs PC Disinhibited characteristics and binge drinking among university student drinkers Addict Behav 35 3 242 251 10.1016/j.addbeh.2009.10.020 19926401

[B9] Van Tieu H Koblin BA HIV, alcohol, and noninjection drug use Curr Opin HIV AIDS 2009 4 4 314 318 10.1097/COH.0b013e32832aa902 19532070

[B10] Kalichman SC Simbayi LC Kaufman M Cain D Jooste S Alcohol use and sexual risks for HIV/AIDS in sub-Saharan Africa: systematic review of empirical findings Prev Sci 2007 8 2 141 151 10.1007/s11121-006-0061-2 17265194

[B11] Fisher JC Bang H Kapiga SH The association between HIV infection and alcohol use: a systematic review and meta-analysis of African studies Sex Transm Dis 2007 34 11 856 863 10.1097/OLQ.0b013e318067b4fd 18049422

[B12] Chersich MF Rees HV Scorgie F Martin G Enhancing global control of alcohol to reduce unsafe sex and HIV in sub-Saharan Africa Global Health 2009 5 16 10.1186/1744-8603-5-16 19919703 PMC2781801

[B13] Chersich MF Luchters SM Malonza IM Mwarogo P King'ola N Temmerman M Heavy episodic drinking among Kenyan female sex workers is associated with unsafe sex, sexual violence and sexually transmitted infections Int J STD AIDS 2007 18 11 764 769 10.1258/095646207782212342 18005511

[B14] Wechsberg WM Luseno WK Lam WK Parry CD Morojele NK Substance Use, Sexual Risk, and Violence: HIV Prevention Intervention with Sex Workers in Pretoria AIDS Behav 2006 10 2 131 137 10.1007/s10461-005-9036-8 16482408

[B15] Parry CD Carney T Petersen P Dewing S Needle R HIV-risk behavior among injecting or non-injecting drug users in Cape Town, Pretoria, and Durban, South Africa Subst Use Misuse 2009 44 6 886 904 10.1080/10826080802487028 19444728

[B16] Browne FA Wechsberg WM The intersecting risks of substance use and HIV risk among substance-using South African men and women Curr Opin Psychiatry 10.1097/YCO.0b013e32833864eb PMC3784346 20308902

[B17] Rehm J Baliunas D Borges GL Graham K Irving H Kehoe T Parry CD Patra J Popova S Poznyak V The relation between different dimensions of alcohol consumption and burden of disease: an overview Addiction 2010 105 5 817 843 10.1111/j.1360-0443.2010.02899.x 20331573 PMC3306013

[B18] WHO Global Information System on Alcohol and Health 2011

[B19] WHO Global status report on alcohol and health 2011

[B20] Lane T Raymond HF Dladla S Rasethe J Struthers H McFarland W McIntyre J High HIV Prevalence Among Men Who have Sex with Men in Soweto, South Africa: Results from the Soweto Men's Study AIDS Behav 2009 10.1007/s10461-009-9598-y PMC2888758 19662523

[B21] Lane T Shade SB McIntyre J Morin SF Alcohol and sexual risk behavior among men who have sex with men in South african township communities AIDS Behav 2008 12 4 Suppl S78 85 18392672 10.1007/s10461-008-9389-x

[B22] Parry C Petersen P Dewing S Carney T Needle R Kroeger K Treger L Rapid assessment of drug-related HIV risk among men who have sex with men in three South African cities Drug Alcohol Depend 2008 95 1-2 45 53 10.1016/j.drugalcdep.2007.12.005 18242881

[B23] Geibel S Luchters S King'Ola N Esu-Williams E Rinyiru A Tun W Factors associated with self-reported unprotected anal sex among male sex workers in Mombasa, Kenya Sex Transm Dis 2008 35 8 746 752 10.1097/OLQ.0b013e318170589d 18650772

[B24] Baggaley RF White RG Boily MC HIV transmission risk through anal intercourse: systematic review, meta-analysis and implications for HIV prevention Int J Epidemiol 2010 10.1093/ije/dyq057 PMC2929353 20406794

[B25] A U D I T. The Alcohol Use Disorders Identification Test. Guidelines for Use in Primary Care http://whqlibdoc.who.int/hq/2001/WHO_MSD_MSB_01.6a.pdf

[B26] Geibel S van der Elst EM King'ola N Luchters S Davies A Getambu EM Peshu N Graham SM McClelland RS Sanders EJ 'Are you on the market?': a capture-recapture enumeration of men who sell sex to men in and around Mombasa, Kenya AIDS 2007 21 10 1349 1354 10.1097/QAD.0b013e328017f843 17545712

[B27] Saunders JB Aasland OG Babor TF de la Fuente JR Grant M Development of the Alcohol Use Disorders Identification Test (AUDIT): WHO Collaborative Project on Early Detection of Persons with Harmful Alcohol Consumption--II Addiction 1993 88 6 791 804 10.1111/j.1360-0443.1993.tb02093.x 8329970

[B28] WHO Sexually Transmitted and Other Reproductive Tract Infections: A guide to essential practice 2005

[B29] Armitage B Berry G Matthews JNS Statistical methods in medical research. Blackwell publishing 2002

[B30] Rehm J Rehn N Room R Monteiro M Gmel G Jernigan D Frick U The global distribution of average volume of alcohol consumption and patterns of drinking Eur Addict Res 2003 9 4 147 156 10.1159/000072221 12970583

[B31] Surveys of drinking patterns and problems in seven developing countries. WHO monograph on alcohol epidemiology in developing countries http://www.unicri.it/min.san.bollettino/dati/AlcBrochur.pdf

[B32] Peltzer K Prevalence of alcohol use by rural primary care outpatients in South Africa Psychol Rep 2006 99 1 176 178 10.2466/pr0.99.1.176-178 17037464

[B33] Peltzer K Seoka P Mashego TA Prevalence of alcohol use in a rural South African community Psychol Rep 2004 95 2 705 706 10.2466/pr0.95.2.705-706 15587239

[B34] Kaner E Bland M Cassidy P Coulton S Deluca P Drummond C Gilvarry E Godfrey C Heather N Myles J Screening and brief interventions for hazardous and harmful alcohol use in primary care: a cluster randomised controlled trial protocol BMC Public Health 2009 9 287 10.1186/1471-2458-9-287 19664255 PMC2734851

[B35] Kaner EF Beyer F Dickinson HO Pienaar E Campbell F Schlesinger C Heather N Saunders J Burnand B Effectiveness of brief alcohol interventions in primary care populations Cochrane Database Syst Rev 2007 2 CD004148 10.1002/14651858.CD004148.pub3 17443541

[B36] Fisher A Foreit J Designing HIV/AIDS Intervention Studies: An Operations Research Handbook. Population Council, Washington 2002

[B37] Weinhardt LS Carey MP Carey KB Maisto SA Gordon CM The relation of alcohol use to HIV-risk sexual behavior among adults with a severe and persistent mental illness J Consult Clin Psychol 2001 69 1 77 84 11302280 10.1037//0022-006x.69.1.77 PMC2424204

[B38] International guide for monitoring alcohol consumption and related harm http://whqlibdoc.who.int/hq/2000/WHO_MSD_MSB_00.4.pdf

[B39] McIntyre JA The need for HIV prevention interventions for men who have sex with men in Africa Sex Transm Infect 2010 86 2 82 83 10.1136/sti.2009.041640 20332365 PMC2923280

[B40] Baral S Sifakis F Cleghorn F Beyrer C Elevated risk for HIV infection among men who have sex with men in low-and middle-income countries 2000-2006: a systematic review PLoS Med 2007 4 12 e339 10.1371/journal.pmed.0040339 18052602 PMC2100144

[B41] McIntyre JA The need for HIV prevention interventions for men who have sex with men in Africa Sex Transm Infect 86 2 82 83 10.1136/sti.2009.041640 PMC2923280 20332365

[B42] Fritz K Morojele N Kalichman S Alcohol: the forgotten drug in HIV/AIDS Lancet 2010 376 9739 398 400 10.1016/S0140-6736(10)60884-7 20650516 PMC3015091

